# Professional Support After Partner Loss: Likelihood and Correlates of Help-Seeking Behavior

**DOI:** 10.3389/fpsyg.2021.767794

**Published:** 2021-11-23

**Authors:** Daniela S. Jopp, Charikleia Lampraki, Claudia Meystre, Hansjörg Znoj, Jeannette Brodbeck

**Affiliations:** ^1^Institute of Psychology, University of Lausanne, Lausanne, Switzerland; ^2^LIVES Center, Swiss Centre of Expertise in Life Course Research, Lausanne, Switzerland; ^3^Institute of Psychology University of Bern, Bern, Switzerland

**Keywords:** help seeking behavior, widowhood, separation, divorce, professional support

## Abstract

Intimate partner loss in later life can be one of the most stressful events in adulthood. Individuals who struggle to adapt to the new life conditions may need support from a mental health professional. However, less is known about the likelihood to seek professional help after separation, divorce, or bereavement in later life and associated factors. This study investigated professional help-seeking (PHS) for partner loss after a long-term marriage in separated, divorced, and bereaved individuals and examined the extent to which specific person and event-related variables, as well as depressive symptoms, increase its likelihood. The data were derived from the LIVES “Intimate Partner Loss Study.” The self-administered questionnaires were completed by 388 adults. PHS was higher after separation (57%) and divorce (49%), compared to widowhood (18%). Higher likelihood of PHS was associated with separation and divorce, female gender, having someone to count on, loss unexpectedness, needing more time to overcome the loss, and more depressive symptoms. Informing individuals unlikely to seek help (e.g., males, bereaved, and individuals with no confidant) about PHS benefits may facilitate adaptation to partner loss.

## Introduction

Losing their spouse, either through divorce or death, represents a critical life event with substantial consequences in many key life domains for most individuals (Amato, 2000; Utz et al., 2012; Lin et al., 2019). Although divorce and bereavement clearly differ in nature, their effects are similarly dramatic: Compared to married individuals, bereaved or divorced individuals have more chronic diseases, more mobility limitations, lower subjective health, and poorer life satisfaction as well as a higher risk of mortality and depression (Lucas, 2005; Stroebe et al., 2007; Hughes and Waite, 2009; Staehelin et al., 2012; Browning et al., 2015; Ory and Huijts, 2015). At the same time, research also shows that about half of the individuals confronted with partner loss follow a resilient trajectory of psychological adaptation, that is, they show less reduction in functionality, or do so only over a short period of time (Booth and Amato, 1991; Bonanno et al., 2002). The other half, however, need longer for recovery (about 1.5–2 years) or may even suffer from a chronic reduction in functioning (Bennett et al., 2020). Thus, given that 16,210 individuals experienced divorce and 28,361 experienced bereavement in 2020 in Switzerland, a small country with over 8,000,000 citizens [Swiss Federal Office of Statistics (SFOS), 2021], a substantial number of people may have more difficulty with recovery. Helping these less resilient individuals to better adapt would be an important public health goal, and professional support *via* healthcare professionals, counselors or psychologists may enable individuals to more quickly accommodate and integrate loss into their lives. Still, professional help-seeking (PHS) after partner loss is not well understood, including which person or event-specific variables increase or reduce the probability of seeking out professional help. In this study, we compared separated, divorced, and bereaved individuals aged 40–91 years regarding their likelihood of PHS in order to identify the frequency as well as the correlates of PHS. While all groups share the experience of partner loss, the odds for PHS may depend on the event type. Taking all three groups into account allows us to compare the likelihood of PHS across them and to consider whether different correlates of PHS are involved.

While the negative effects of partner loss are supported by substantial empirical evidence, a bulk of studies also indicates that specific person- (e.g., demographic and psychological aspects), relationship- (e.g., prior relationship quality), and event-related aspects (e.g., circumstances of partner loss) modulate the extent to which the event is experienced as disruptive (Thuen and Rise, 2006; Spahni et al., 2015; Knöpfli et al., 2016). For instance, divorced/separated individuals coming from unsatisfying and conflictual marriages are no more likely than married individuals to develop a mental disorder (Overbeek et al., 2006). In contrast, widowed individuals who reported that their marriage had been harmonious were found to have the highest healthcare costs compared to married and bereaved with low marital harmony (Prigerson et al., 2000). Widow(er)s have been also found to have an increased risk for health deterioration and mortality in the first months after spousal loss (Seiler et al., 2020). Social support plays an important role in the adaptation process: For bereaved individuals, most social support was provided in the first month post-loss (Powers et al., 2014), and it was associated with fewer depressive symptoms (Pudrovska and Carr, 2008). For divorcees, having someone to count on for help and a new partner was related to the lower levels of social loneliness (Lampraki et al., 2019). Ultimately, finding a new partner makes a big difference: According to Bowen and Jensen (2017), re-partnered divorcees and widow(er)s were feeling more satisfied with life than unpartnered divorcees or widow(er)s. Lin and colleagues also identified that widowed and divorced individuals who re-partnered after spousal loss had fluctuations in depressive symptoms, which finally stabilized after several years at the pre-loss levels.

An important person variable playing a role for adaptation to partner loss may be the chronological age. Overall, the number of older individuals affected by bereavement or divorce has grown, with both partner loss events being likely to happen later in the life course, probably due to the increase in life expectancy: For individuals aged 80 years and older, widowhood showed a 30% rise in Switzerland between 1999 and 2019 and a 20% rise for 65- to 79-year-olds [Swiss Federal Office of Statistics (SFOS), 2021]. During the same period, the number of divorcees increased by 40% among those aged 50–59 years old in Switzerland and doubled for those over 60 years of age [Swiss Federal Office of Statistics (SFOS), 2021]. A similar development, discussed as “gray divorce revolution”, has been described for other countries, including the United States, where the divorce rate doubled between 1990 and 2010 for individuals aged 50 years and older (Brown and Lin, 2012; Lin and Brown, 2020).

Compared to younger people, older individuals who lost their partner may be more vulnerable due to biological, social, and psychological age-related aspects (Perrig-Chiello et al., 2015), such as physical changes (e.g., health issues and menopause), multiple social roles (e.g., being a mother and caregiving to frail parents), and feeling unable to re-partner (e.g., length of the previous relationship and lack of potential partners). Coping resources may also be reduced: Due to their stronger focus on family relationships (Fuller et al., 2020), older individuals may have more social support from family but may also suffer more from family disruption due to the loss of the spouse. Given their potentially increased vulnerability, older individuals may benefit especially from professional help, but only a few studies focus on this age group.

A large amount of information is available on mental or physical health problems associated with partner loss; however, research on how these negative consequences translate into health service use is limited. Research evidence is mixed regarding whether divorce or bereavement is more or less difficult to overcome (Luhmann et al., 2012), but the study by Papa et al. (2014) suggested that the adaptation to both divorce and bereavement is, at least up to 1 year after the event, characterized by similar challenges, which could be reduced by professional help.

The limited evidence available at present suggests that divorced individuals are more likely to seek professional help. For instance, a large European study (*N* = 27,146) showed that divorced or separated individuals had higher PHS than married/cohabiting individuals (Bracke et al., 2010), even when controlling for need (e.g., mental health) and enabling factors (e.g., family or friends as first health contacts). In a smaller Belgian sample, however, only divorced women without a new partner had higher PHS and also reported more unmet care needs compared to divorced women with partners, divorced men, and married individuals (Colman et al., 2012).

Bracke et al. (2010) also found that the physical and mental health status only partially accounted for PHS after separation or divorce, whereas in widowhood, mental health status explained interindividual differences in PHS. This was in line with earlier findings from the Americans’ Changing Lives Survey investigating 927 women (*M*_*Age*_ = 52 years), which showed that widowhood was related to higher depression levels and poorer physical health but not to increased service use (i.e., physical and mental), while separation and divorce were not associated with depression or physical health but with more mental health service use (Prigerson et al., 1999). Thus, while divorced and separated individuals may seek professional help for specific issues linked to the marital breakup such as intrusive thoughts, bitterness, or practical issues (e.g., childcare conflicts), PHS may be a less common practice among the widowed. This may also be due to the fact that widowed individuals are likely to be older and thus less attuned to request mental health services. So far, findings are quite inconclusive as the PHS of widow(er)s varies widely among studies, ranging from 3.7% (Currow et al., 2008) to 11.5% (Caserta and Lund, 1992).

In the context of PHS, there is an important need to understand which characteristics of the person or the event may facilitate or hinder PHS and to increase the likelihood that individuals in need receive appropriate support. Several studies undertaken in the broader context of mental illness have shown sociodemographic aspects to predict PHS, such as older age, female gender, higher financial adequacy, access to health insurance, and higher education (Mojtabai et al., 2002; Wang et al., 2005; Schomerus et al., 2013). For PHS after partner loss, the mentioned European study by Bracke et al. (2010) found that those who were of younger age, female, a student, or retired were all associated with higher PHS. Furthermore, they found that individuals able to nominate a friend (but not a family member) as first health contact showed higher PHS, suggesting that friends may encourage professional support-seeking and thus have a specific enabling role.

Studies further indicate that PHS is associated with event-related aspects. Specifically, individuals were more likely to seek professional help from a psychologist or counselor when experiencing higher emotional distress (Cramer, 1999; Schomerus et al., 2013). In the context of partner loss, the event-related variables can be defined as linked to the loss circumstances and the relationship with the former partner. The studies on adjustment to separation or divorce underline that the unexpectedness of loss and the persistent attachment to the former partner are associated with poorer psychological adaptation (Alain and Lussier, 1988; Halford and Sweeper, 2013). These studies have, however, not investigated the links to PHS. For bereavement, factors predicting PHS were strong attachment to the deceased (e.g., having provided caregiving), higher distress (i.e., higher depression and low subjective health), and poor coping capacity (e.g., being unable to “move on,” Caserta and Lund, 1992; Currow et al., 2008).

This study investigated the likelihood and correlates of PHS (e.g., professional support provided by medical doctors and psychologists) among individuals with different types of partner loss. A few research has dealt with this topic, and the available results suggest potential differences in PHS frequency among separated, divorced, and widowed individuals. However, these studies investigated only to a limited extent, or not at all, which person and event-related variables could facilitate or hinder PHS after partner loss and attenuate the links between event type and PHS.

To fill this gap, we investigated whether separated, divorced, and widowed individuals differed in PHS in a sample of Swiss individuals aged 40 years and older. We focused on individuals who had lost their partner up to 24 months prior to study participation, as this time frame represents the main adaptation phase to partner loss according to the past research (Booth and Amato, 1991; Bonanno et al., 2002; Lucas, 2005; Pudrovska and Carr, 2008). To gain a clearer picture of the individuals seeking professional help after partner loss, we examined the importance of an expanded set of person variables for PHS. In line with prior research (Bracke et al., 2010; Schomerus et al., 2013), we expected that being younger, female, and having a better financial situation would increase the likelihood of PHS, while having a new partner would reduce the likelihood of PHS.

As the event-related predictors of PHS have rarely been addressed before, with a handful of studies investigating these in the context of bereavement, we also expanded on this research by adding variables likely to play a role. In line with prior studies (Alain and Lussier, 1988; Currow et al., 2008; Halford and Sweeper, 2013), we compared the specific types of partner loss (separation vs. divorce vs. widowhood) with respect to their likelihood of higher PHS and hypothesized that bereaved individuals would be less likely to seek professional help than the other two groups, although we expected that other event-related variables might attenuate this finding. For the other event-related predictors, we assumed that shorter time since the loss, unexpectedness of the loss, and the feeling of not being able to psychologically overcome the loss would increase the likelihood of PHS; in line with the findings by Bracke et al. (2010), we further expected that having more social support during the crisis (i.e., a confidant helping to better cope with the loss) would increase the likelihood of PHS. Finally, we hypothesized that individuals with worse mental health after partner loss, specifically with more depressive symptoms, would be more likely to seek PHS.

## Materials and Methods

### Sample and Study Procedures

For this study, we focused on individuals who experienced separation, divorce, or bereavement during the past 2 years, who were at least 40 years of age, and married for at least 10 years before the loss. Our sample was part of a larger representative study investigating the adaptation to partner loss of individuals in the second half of life in Switzerland (Knöpfli et al., 2016). The majority of the participants of the larger study was based on individuals who had been randomly drawn by the Federal Statistical Office (BFS, 2011), stratified by age group, gender, and marital status; the present subsample instead was mainly recruited *via* advertisement. This was due to the fact that the Federal Statistical Office, in an effort to protect individuals undergoing partner loss transitions, does not offer information on recent bereavement and divorce (i.e., individuals who have experienced both events within the past 24 months are listed as married). Contacting these participants led to identifying some recently bereaved or divorced individuals, but this group was too small compared with the rest of the representative sample, thus requiring enrichment through advertising. It is also of note that, in Switzerland, individuals who wish to divorce have to stay legally separated for 2 years before they can finalize the dissolution of the marriage.

The final sample included a total of *N* = 388 participants (73% of women), with a mean age of 55.58 years (*SD* = 10.50; age range 40–91). Of these, 152 (39%) were separated, 149 (38%) were divorced, and 87 (23%) were widowed (Table 1). Participants filled out a questionnaire at home, utilizing either a paper-and-pencil version or a web-based online version.

**TABLE 1:** Person and event-related characteristics and professional help-seeking (PHS) of the total sample and by the critical marital event.

	**Total (*N* = 388)**		**Separation (*n* = 152)**		**Divorce (*n* = 149)**		**Widowhood (*n* = 87)**				
	***M* or *N* (*SD* or *%*)**	**Range**	***M* or *n* (*SD* or *%*)**	**Range**	***M* or *n* (*SD* or *%*)**	**Range**	***M* or *n* (*SD* or *%*)**	**Range**	***F* or χ *2***	***n, df***	**ϕ or η *^2^***
Age	55.58 (10.50)	40–91	50.90 (7.32)	40–79	53.32 (7.49)	41–79	67.62 (10.53)	40–91	123.94***	387, 2	0.40
Gender (men)	103 (27)		32 (21)		51 (34)		20 (23)		7.43*	388,2	0.14
Education									4.54	388, 6	0.11
Compulsory school	16 (4)		6 (4)		6 (4)		4 (5)				
Professional formation and high school	185 (48)		67 (44)		69 (46)		49 (56)				
Technical college	124 (32)		50 (33)		50 (34)		24 (28)				
University	63 (16)		29 (19)		24 (16)		10 (12)				
Income adequacy									18.18**	388, 4	0.22
Not enough money	50 (13)		27 (18)		21 (14)		2 (2)				
Enough money	292 (75)		107 (70)		117 (79)		68 (78)				
More than enough money	46 (12)		18 (12)		11 (7)		17 (20)				
Time since event	1.24 (0.72)	0–2	1.25 (0.71)	0–2	1.20 (0.76)	0–2	1.31 (0.65)	0–2	0.73	387, 2	0.004
Re-partnered (yes)	85 (22)		27 (18)		52 (35)		6 (7)		25.72***	380, 2	0.26
Person to count on (yes)	335 (89)		135 (89)		121 (84)		79 (95)		6.65*	378, 2	0.13
Unexpectedness of loss (yes)	161 (42)		70 (46)		57 (38)		34 (39)		2.15	388, 2	0.08
Time to overcome the loss									49.45***	388, 4	0.36
Less than 1 year	65 (17)		24 (16)		33 (22)		8 (9)				
More than 1 year	102 (26)		26 (17)		63 (42)		13 (15)				
Never	221 (57)		102 (67)		53 (36)		66 (76)				
Depressive symptoms	0.84 (0.61)	0–3	0.95 (0.67)	0–3	0.70 (0.58)	0–3	0.87 (0.53)	0–3	5.81**	346, 2	0.03
Professional help-seeking (yes)	176 (45)		87 (57)		73 (49)		16 (18)		34.98***	388, 2	0.30

Gender: 1 = female, 2 = male. Re-partnered: 1 = yes, 0 = no. Unexpected loss: 1 = yes, 0 = no. PHS: 1 = yes, 0 = no. *p < 0.05; **p < 0.01; ***p < 0.001.

### Measures

#### Professional Help-Seeking

PHS was measured with the question “What did you do after the separation/divorce/bereavement to cope with the new situation?” and one of the proposed answers among others (e.g., seek help from friends) was “seek help from a professional, e.g., doctor, psychologist,” with an answering format of 0 = *no* and 1 = *yes*.

#### Person Characteristics

As person variables, we included age (in years), gender (1 = male and 2 = female), educational level (1 = compulsory school; 2 = professional formation/high school; 3 = technical college; and 4 = university), and income adequacy (1 = not having enough money to meet the needs of an individual; 2 = having enough money to meet the needs of an individual; and 3 = having more than enough money to meet the needs of an individual).

#### Event-Related Characteristics

The *type of event* was assessed with the question: “During your life, did you ever lose a long-term partner through separation, divorce, or death?” Participants answered by *yes* (=1) or *no* (=0) to the three propositions, reporting also the year of the event; only those having experienced the event in the past 2 years and having been married for at least 10 years before the loss were retained in the analysis. *Time since the loss* was also reported in years.

*Social resources* included whether the person had *re-partnered* since the loss (“Are you currently in a relationship?”: 0 = *no*; 1 = *yes*) and if the person had *someone to count on* when the loss occurred [“Were you able to count on the help of someone to deal better with the separation/divorce/death of your spouse? (family, friends, others—except health professionals)”: 0 = *no*; 1 = *yes*].

Additional *event-specific characteristics* included *unexpectedness* (“Was the separation/the death of your partner unexpected?”: 0 = *no* or *not completely*; 1 = *yes*), and *time to overcome the loss* (“How long did it take you to psychologically overcome this breakup/this loss?”: 0 = less than 1 year; 1 = more than 1 year; 2 = not enough time has passed to get over it; 3 = I will probably never really psychologically overcome this separation). For the following analysis, the latter two categories were combined.

*Depressive symptoms* were measured with the Center for Epidemiological Studies Depression Scale (Radloff, 1977). The scale comprised of 15 items (e.g., I am feeling sad) and the answering format ranged from 0 = rarely/not at all to 3 = most of the time/all the time. A composite mean score was created (Cronbach’s α = 0.92).

### Statistical Analyses

We used cross-tabulations with χ^2^ tests and ANOVA (*F*-test) to analyze the differences between separated, divorced, and widowed participants regarding the person and event-related characteristics, and PHS. We then ran a multiple hierarchical logistic regression analysis to examine whether PHS after partner loss was associated with the specific person and event-related characteristics, using a stepwise procedure. We selected all independent variables *a priori* based on the literature and included them in steps in the model: First, we included the person’s characteristics (i.e., age, gender, education, and financial situation; Step 1). Then, we included event-specific characteristics, splitting them up into several steps: first, the event type and time since the loss variables (Step 2), followed by social resources (i.e., re-partnered and someone to count on; Step 3) and more psychological event-specific characteristics (i.e., unexpectedness and time to overcome the loss; Step 4), and, finally, by depressive symptoms (Step 5). As we were interested in examining whether the effect of the specific person or event-related characteristics had a differential impact on PHS depending on the type of loss, we also tested the interactions between the type of event (separated vs. divorced vs. bereaved) and all other independent variables. Interaction testing was performed in separate analyses (i.e., main model plus one interaction each time, to avoid overpowering the model). However, none of the interactions was significant. Therefore, we reported only the model with the main effects. Notably, we would talk about “predictors” and “prediction” of interindividual variance in the context of regression models, referring to statistical terminology without implying causality.

## Results

### Comparison of Separated, Divorced, and Widowed Individuals Regarding the Person and Event-Related Variables, and Professional Help-Seeking

Separated, divorced, and widowed individuals differed with respect to several characteristics (Table 1). Widowed participants were significantly older (*M* = 67.62 years) than separated (*M* = 50.90 years) and divorced individuals (*M* = 53.32 years; *F* = 123.94; *p* < 0.001; η^2^ = 0.40). Considering the gender distributions, men represented a larger part within the divorced group (34%), in comparison with the other two groups (21% in separated and 23% in widowed; χ^2^ = 7.43, *p* < 0.05; ϕ = 0.14). While groups did not differ in education, they did in financial adequacy: compared to separated (13%) and divorced individuals (18%), the bereaved were more satisfied with their financial situation, with only 2% of them reporting not having enough money to support their needs (χ^2^ = 18.18, *p* < 0.001; ϕ = 0.22). Regarding re-partnering, 18% of the separated and 35% of the divorced individuals reported having found a new partner, while the bereaved re-partnered less often (7% with a new partner; χ^2^ = 25.72, *p* < 0.001; ϕ = 0.26). Widow(er)s were more likely to have someone to count on for helping them deal with the loss (95%) than the separated (89%) and divorced individuals (84%; χ^2^ = 6.65, *p* < 0.05; ϕ = 0.13). The strongest difference between groups was found for time to psychologically overcome the loss (ϕ = 0.36): Fewer divorcees (36%) reported that they did not have or would never overcome the loss of the partner compared to the separated (67%) and the widowed individuals (76%; χ^2^ = 49.45, *p* < 0.001). Divorcees experienced fewer depressive symptoms (*M* = 0.70) in comparison with the separated (*M* = 0.95) and widowed groups (*M* = 0.87), with separated feeling worse in terms of depressive symptoms (*F* = 5.81; *p* < 0.01; η^2^ = 0.03). Finally, about half of the separated (57%) and divorced (49%) but only 18% of the widowed participants sought professional help. This difference was significant (χ^2^ = 34.98, *p* < 0.001) with a moderate effect size (*ϕ* = 0.30).

By presenting the bivariate correlations, Table 2 complements the findings reported earlier: PHS was associated with younger age, being a woman, having someone to count on to deal with the loss, experiencing the loss as unexpected, needing more time to overcome the loss, and more depressive symptoms. PHS was marginally positively linked to income adequacy. In addition, time passed since the event was negatively correlated with more time needed to overcome the loss; the closer the event objectively was, the more likely the person was to indicate that they had not yet or would never overcome the loss. Having a new partner was related to younger age, being a man, being more educated, not having expected the loss, and needing less time to overcome the loss. Men were also more likely to not having noticed the separation coming. Needing more time to overcome the loss was related to not having expected the loss and being a woman. Stronger depressive symptoms were associated with being a woman, less educated, having more than enough money to support oneself, being closer to the event, not having a new partner and someone to count on, not expecting the loss, and needing more time to overcome the loss (correlations presented by event type, refer to Supplementary Tables).

**TABLE 2:** Correlations between the person and event-related characteristics and PHS of the total sample (*N* = 388).

	**1**	**2**	**3**	**4**	**5**	**6**	**7**	**8**	**9**	**10**	**11**	**12**
1. Age (years)	1											
2. Gender	0.07	1										
3. Education	−0.12*	0.26***	1									
4. Income adequacy	0.11*	0.04	0.21***	1								
5. Type of event	0.56***	0.04	−0.10^+^	0.16**	1							
6. Time since loss (years)	0.08^+^	–0.04	0.06	0.07	0.02	1						
7. Re-partnered	−0.20***	0.27***	0.11*	0.05	–0.05	0.09^+^	1					
8. Someone to count	0.03	–0.08	0.03	0.06	0.05	–0.04	–0.03	1				
9. Unexpected loss	−0.14**	–0.07	–0.08	–0.01	–0.06	–0.07	−0.12*	0.05	1			
10. Time to overcome the loss	0.08	−0.12*	–0.07	–0.02	0.03	−0.13*	−0.24***	0.02	0.24***	1		
11. Depressive symptoms	–0.01	−0.14*	−0.15**	0.24***	–0.08	−0.12*	−0.29***	−0.12*	0.22***	0.37***	1	
12. Professional help-seeking	−0.20***	−0.18***	0.04	0.09^+^	−0.28***	–0.03	–0.07	0.13*	0.17**	0.20***	0.19***	1

Gender: 1 = female, 2 = male. Education: 1 = compulsory school, 4 = university. Income adequacy: 1 = not enough money, 3 = more than enough money. Re-partnered: 1 = yes, 0 = no. Unexpected loss: 1 = yes, 0 = no. Time to overcome the loss: 1 = less than 1 year, 3 = not enough time/never. PHS: 1 = yes, 0 = no. ^+^*p* < 0.10, *p < 0.05, **p < 0.01, ***p < 0.001.

### Person, Event-Related Characteristics, and Depressive Symptoms Associated With Professional Help-Seeking

We conducted a stepwise hierarchical logistic regression to determine the likelihood of PHS considering person and event-related variables. As explained in Table 3, adding variables in later steps did not lead to reducing the significance or size of effects found in earlier steps. In contrast, effects became stronger when considering more variables, with two exceptions described later. Thus, we will concentrate on describing the findings for the full model (Step 5). The full model was statistically significant, χ^2^ (14, *N* = 388) = 93.47, *p* < 0.001, indicating that it was able to distinguish between participants who had vs. those who had not sought professional help. It explained 33% (Nagelkerke *R*^2^) of the variance in PHS and correctly classified 72.8% of cases. Six of the independent variables made a statistically significant (or marginal) contribution to predicting the likelihood of PHS (i.e., gender, type of event, having someone to count on, unexpectedness, time to overcome the loss, and depressive symptoms). Specifically, the strongest predictor of PHS was the type of event showing that separated individuals were almost 11 times [odds ratio (OR) = 10.57] more likely and divorcees were almost 10 times (OR = 9.83) more likely to seek professional help than widowed individuals. Among the person variables, only gender was significant: men were less likely to seek professional help than women (OR = 0.42). While having a new partner was not associated with PHS, having someone to count on for dealing with the loss increased the likelihood of PHS by almost four times (OR = 3.74) compared to having nobody to count on. Not having expected the loss was also marginally related to an almost two times (OR = 1.60) higher likelihood of PHS than when the loss was expected. It was also notable that while time since the event was not a significant predictor, subjective time to overcome the loss was related to PHS: individuals indicating that they needed more than 1 year to overcome the loss (OR = 2.31) or would never overcome it (OR = 3.62) were, respectively, two and almost four times more likely to seek help. Finally, individuals who experienced depressive symptoms after the loss were almost two times (OR = 1.73) as likely to seek help.

**TABLE 3:** Person and event-related characteristics associated with PHS after partner loss (*N* = 388).

	**Step 1**		**Step 2**		**Step 3**		**Step 4**		**Step 5**	
	**B**	**Odds Ratio**	**B**	**Odds Ratio**	**B**	**Odds Ratio**	**B**	**Odds Ratio**	**B**	**Odds Ratio**
Age	−0.04**	0.96	–0.0004	1.00	–0.02	1.00	0.002	1.00	0.002	1.00
Gender (*men*)	−0.86**	0.42	−1.03***	0.36	−0.93**	0.40	−0.89**	0.41	−0.90**	0.42
Education	0.22	1.24	0.18	1.20	0.15	1.16	0.21	1.24	0.24	1.28
**Income adequacy (reference: *Not having enough money)***										
Enough money	–0.69	0.50	–0.23	0.80	–0.26	0.77	–0.39	0.68	–0.12	0.89
More than enough money	–0.54	0.58	–0.42	0.66	–0.48	0.62	–0.44	0.64	–0.22	0.81
**Type of event (reference: *Bereavement*)**										
Divorce			2.06***	7.85	2.15***	8.55	2.29***	9.85	2.29***	9.83
Separation			1.74***	5.72	1.96***	7.11	2.33***	10.23	2.36***	10.57
Time since the loss			–0.05	0.96	0.02	1.02	0.09	1.10	0.10	1.11
Re-partnered (*yes*)					–0.36	0.70	–0.09	0.91	0.04	1.04
Someone to count on (*yes*)					1.09**	2.98	1.17**	3.21	1.32**	3.74
Unexpectedness (*yes*)							0.57*	1.78	0.47^+^	1.60
**Time to overcome the loss (reference: *Less than 1 year*)**										
More than 1 year							0.85*	2.33	0.84^+^	2.31
Not enough/Never							1.45***	4.27	1.29**	3.62
Depressive symptoms									0.55*	1.73
Nagelkerke *R*^2^	0.11		0.20		0.23		0.31		0.33	

*Gender: 1 = female, 2 = male. Education: 1 = compulsory school, 4 = university. Income adequacy: 1 = not enough money, 3 = more than enough money. Re-partnered: 1 = yes, 0 = no. Unexpected loss: 1 = yes, 0 = no.*

Time to overcome the loss: 1 = less than 1 year, 3 = not enough time/never. PHS: 1 = yes, 0 = no. +*p* < 0.10; *p < 0.05; **p < 0.01; ***p < 0.001.

The stepwise procedure, as depicted in Table 3, shows that the person and event-related variables predicted PHS independently from each other, with the exception of age and unexpected loss: when considering basic characteristics only (Step 1), age was a significant predictor of PHS, with higher age being associated with a lower PHS probability (OR = 0.96). The effect of age disappeared when entering the key event-related variables, i.e., type of event and time since the loss (Step 2). Type of event was significant, whereas time since the event was not. Consequently, younger individuals had a greater likelihood of PHS only because they were more likely to undergo separation and divorce than older individuals. Similarly, in Step 4, the unexpectedness of the loss was significantly related to PHS, showing that, when the loss was not expected, the individual was more likely to seek professional help (OR = 1.78). Then, in Step 5, when the depressive symptoms were added in the model, the unexpectedness of the loss became marginally related to PHS (OR = 1.60).

## Discussion

This study investigated PHS after separation, divorce, or bereavement and analyzed which person or event-related variables facilitated or hindered PHS. We found that almost two-thirds of the separated and half of the divorced participants used professional help, compared to only about one-fifth of the widowed. When controlling for the person and event-related factors, separated and divorced individuals were 11 and 10 times, respectively, more likely to seek professional help than widowed individuals.

That separated and divorced are more likely to seek out professional help than bereaved individuals is in line with prior studies (Prigerson et al., 1999; Bracke et al., 2010), yet our findings revealed much stronger effects and added clarity by controlling for confounding variables such as age. As underlined by Bracke et al. (2010), separated or divorced individuals may want to understand why their relationship ended and how they could improve their way of being and acting for future partnerships, as they may still hope to find a new partner. Therefore, they may seek support to work on issues related to the past partnership, to facilitate a new relationship. Instead, bereaved individuals may feel less need to rework partnership issues, which could contribute to less PHS.

Moreover, the attribution of the marital event might be different in the case of separation/divorce vs. bereavement. Some separated and divorced individuals may feel responsible for the end of the relationship, while this is less likely in the case of the death of the partner. Such internal attributions may relate to more PHS, as shown, for instance, in a study on the use of mothers of a family support program (Telleen, 1990). Similarly, “disenfranchised grief” (grief that is not socially acknowledged), as proposed by Doka (2002), may play a role in seeking professional help; in comparison with widow(er)s, separated and divorced individuals may more often experience a similar lack of recognition, if family and friends do not acknowledge their pain and mourning process. Separated individuals were the group with the highest depressive symptoms, which could be associated with this lack of recognition; besides, the depression levels could also be an indicator of closeness to the actual life event and strong ongoing adaptation efforts. Finally, separated and divorced individuals may seek professional help more often as their network may choose sides and thus represent a more limited source of support than in the case of bereavement. In line with this assumption, a significantly higher percentage of the bereaved in our study indicated having a person to count on, compared to separated or divorced individuals.

Less than one-fifth of bereaved individuals sought out professional help. This frequency is higher than previously reported (e.g., 3.7%: Currow et al., 2008; or 11.5%: Caserta and Lund, 1992), which could be related to selection bias: maybe bereaved individuals with more adaptation difficulty were more interested in study participation. Another reason could be the age of the sample, as we had a particular interest in older individuals; however, as demonstrated earlier, age showed no association with PHS once the type of event had been controlled for.

The PHS being sought out by only every fifth bereaved person may owe to established cultural and religious rituals and grieving practices which facilitate coping with the loss, potentially reducing the need for professional help. Bracke et al. (2010), for example, showed that widowhood was not linked to increased health service use compared to married individuals. A recent meta-analysis found the prevalence of prolonged grief disorders in bereaved adults to be around 10% (Lundorff et al., 2017), which may suggest that the need for psychotherapeutic interventions after the spousal death is indeed non-systematic. In addition, while many widow(er)s are able to overcome their loss based on their own capacities without developing pathology, there are clear variations in how well people adapt and in which time frame; thus, low-level interventions may help to facilitate this process.

Notably, there are also interesting similarities among the separated and divorced regarding PHS, as one could have expected more differences. Although, according to raw data, the frequency of PHS was smaller for the divorced than for the separated—probably because the divorced had more time to adapt to their loss as Swiss legislation requires 2 years of separation before being able to file for divorce—the regression coefficients showed that both separated and divorced did not differ in their likelihood to seek PHS when controlling for other factors including time since the event. Nevertheless, given the legal requirements, one could have expected divorced individuals to have a lower likelihood of PHS compared to the separated, as they already have passed the obligatory separation phase. Still, both phases may offer specific challenges: separated individuals may feel closer to the actual breakup and face challenges related to everyday life, such as finding a new home, adjusting family routines, or struggling with loneliness, which could lead to seeking professional support to deal with these issues. Filing for divorce after 2 years of legal separation entangles other, more formal challenges such as child custody or decisions on common property and fortune that add to the stresses faced during the separation period, prolonging their mental health struggle.

Results on the person and event-related variables expanded prior studies by applying them to be separated, divorced, and bereaved, as well as allowed to control for a larger set of aspects that had not been taken into account before for all three groups. In addition, findings show that these variables had similar associations with PHS, independent of whether individuals had to cope with separation, divorce, or bereavement, as we found no significant interactions between predictors and event type. More specifically, our findings showed that PHS after partner loss was strongly linked to the time needed to overcome the loss mentally and emotionally but not to the actual chronological time since the event. If participants thought that they would never overcome the loss or if it had already taken them more than a year, they were more likely to see a professional. Time to overcome the loss could be an index of the difficulty to move on, which may be associated with the severity of the disturbance caused by the event. This interpretation is in line with the study by Currow et al. (2008), which found that widow(er)s who felt unable to move on with their life were more likely to seek professional support.

Moreover, parallel to previous studies on adaptation after partner loss, our findings confirmed the importance of the (un)expectedness of the loss. If the event was not expected, individuals were much more likely to see a professional, but this effect became marginal when also including depressive symptoms in the analysis. Unexpectedness may make it more difficult to accept the loss, thus professional help may be more often considered.

Similar to prior studies, we found that having a confidant to discuss the partner loss was associated with a higher likelihood of PHS. This is, to some extent, in line with the “crowding-in” effects found earlier, as reported, for example, by Bracke et al. (2010), where mentioning a friend as first health contact was linked to higher PHS. Thus, PHS does not seem to compensate for the lack of informal social partners, such as family and friends, whose presence encourages PHS, maybe because they offer different insights and facilitate deeper reflections, which are then followed up on a professional.

Concerning person characteristics, in contrast to prior studies (Mojtabai et al., 2002; Burns et al., 2003; Wang et al., 2005), we did not find an association between financial adequacy and PHS. In these earlier studies, higher financial resources had acted as an enabling factor for higher mental health service use (Burns et al., 2003; Wang et al., 2005); similarly, health insurance coverage was associated with mental health care use (Mojtabai et al., 2002). That financial background had no importance in our study may be due to the fact that, in Switzerland, health insurance is compulsory, and professional interventions are, to a larger extent, financially covered. Still, there may be internal barriers such as attitudes toward professional help, which may be more important than financial barriers in a rather conservative society such as Switzerland (Bambauer and Prigerson, 2006); however, such variables were not available in our study.

In contrast to Colman et al. (2012) who found that re-partnering facilitated PHS, having a new partner was not related to PHS in our study, neither at the level of zero-order correlations nor in the more complex analysis. This could be related to cultural factors, as anecdotally, divorce seems understood as a personal failure in conservative Switzerland, with this feeling persisting despite having found a new partner. Nevertheless, it is of note that having found a new partner was still associated with lower depression levels, as well as other event-related variables important for predicting PHS, such as loss unexpectedness and time to psychologically overcome the loss. Thus, it remains unclear why re-partnering showed no influence on PHS in our study.

Our findings further suggest that individuals who are struggling more with the loss, such as those with higher levels of depression, were more likely to seek professional support, independent of all other variables. This result parallels the findings from prior studies in which mental health aspects had been integrated to capture the actual need for support of an individual (Bracke et al., 2010; Colman et al., 2012).

Despite the various strengths of our study (e.g., the novelty of research question, comparison of loss groups, sample size, and a comprehensive set of person and event-related variables), some limitations need consideration. One shortcoming is that our study is not representative of the Swiss population, thus some selection bias may apply. Also, PHS after partner loss was assessed retrospectively. Consequently, we did not have data on the level of psychological distress before help-seeking and were unable to analyze whether the increase in distress level was an important predictor for help-seeking or not. Furthermore, we did not have detailed information about the types of professional help, that is, whether participants met a counselor, psychologist, psychiatrist, or general practitioner. Finally, several variables were only assessed as bivariate indicators, which could have covered potential underlying nuances. This may have contributed to the fact that we did not find differential associative patterns of PHS when testing interaction effects between predictor variables and event type. The relatively modest sample size may have also contributed to us being unable to identify the event-specific prediction patterns of PHS. Finally, individuals may have relied on multiple sources of help (e.g., friends, family, religious institutions, and professionals), which may complement each other. Future research should focus on different patterns of needs and support after separation, divorce, and bereavement to develop interventions tailored to the needs of the individuals. Such studies should also place special emphasis on individuals with higher levels of depression but without professional support. Addressing the treatment gap in separated, divorced, and bereaved individuals represents an important public health challenge, and more studies are needed to better understand why individuals coping with partner loss cannot be reached by professional help (Bracke et al., 2010; Aoun et al., 2015).

## Conclusion

Our findings highlight that PHS is much more common among separated or divorced older adults than among widow(er)s. That at least half of the separated and divorced seek help suggests that they are more in need of professional support, probably because there is less support or a lack of cultural scripts for coping with this type of social loss. That only about one-fifth of the bereaved older adults sought out professional help is also noteworthy: although only 1 in 10 widow(er)s develops prolonged grief disorders, there is still a substantial number of individuals who are not resilient and need much time and effort to overcome partner loss. Given these individual differences in adaptation to bereavement, enhancing the awareness of possible professional support and addressing societal barriers to PHS might be very useful, especially for widowers, given that men seek professional help less often than women. For separated and divorced participants, highlighting the availability of professional help services may be an important public health goal, given the increase in “gray” divorces and potential age-related reduction in support and other coping resources. In addition, promoting informal support and culturally shared practices for coping with separation or divorce, (digital) self-help interventions, and including the offer of mediation focusing on the psychological aspects in legal divorce services may help to prevent more problems and facilitate a quicker and more complete adaptation process.

## Data Availability Statement

The datasets presented in this study can be found in online repositories. The names of the repository/repositories and accession number(s) can be found below: https://forsbase.unil.ch/project/study-public-overview/15141/0/.

## Ethics Statement

The studies involving human participants were reviewed and approved by Ethics Committee of the University of Bern. The patients/participants provided their written informed consent to participate in this study.

## Author Contributions

DJ, CL, CM, HZ, and JB contributed to the conception and design of the study. CL organized the database and performed the statistical analysis. CM wrote a first draft of the manuscript. DJ and CL wrote the sections of the manuscript and fully revised the manuscript. HZ was the primary investigator of the project. All authors contributed to the final version of the manuscript, as well as read and approved the submitted version.

## Conflict of Interest

The authors declare that the research was conducted in the absence of any commercial or financial relationships that could be construed as a potential conflict of interest.

## Publisher’s Note

All claims expressed in this article are solely those of the authors and do not necessarily represent those of their affiliated organizations, or those of the publisher, the editors and the reviewers. Any product that may be evaluated in this article, or claim that may be made by its manufacturer, is not guaranteed or endorsed by the publisher.
