# Supplementary material for: Professional Support After Partner Loss: Likelihood and Correlates of Help-Seeking Behavior
Source: Front Psychol. 2021 Nov 23;12:767794. doi: 10.3389/fpsyg.2021.767794 (PMC8649630; doi:10.3389/fpsyg.2021.767794)
Supplement: Supplementary file 1 [file Data_Sheet_1.docx]

Table A1. Correlations between the person and event-related characteristics, and professional help seeking for the subsamples of separated (*n* = 152; below diagonal) and divorced (*n* = 149; above diagonal)

|  | 1 | 2 | 3 | 4 | 5 | 6 | 7 | 8 | 9 | 10 | 11 |
| --- | --- | --- | --- | --- | --- | --- | --- | --- | --- | --- | --- |
| 1. Age | 1 | .12 | -.03 | .03 | .17 | -.13 | -.06 | -.12 | .06 | .03 | .04 |
| 2. Gender | -.01 | 1 | .17^*^ | -.05 | -.07 | .24^**^ | -.08 | -.04 | -.15^+^ | -.11 | -.14^+^ |
| 3. Education | -.06 | .40^***^ | 1 | .21 | .09 | .09 | .07 | -.04 | -.13 | -.11 | .10 |
| 4. Income adequacy | .01 | .21^*^ | .24^**^ | 1 | .08 | .14^+^ | .15^+^ | .03 | -.05 | .29^***^ | .03 |
| 5. Time since loss | .07 | -.02 | .05 | .04 | 1 | .02 | -.08 | -.08 | -.06 | .06 | -.02 |
| 6. Re-partnered | -.14^+^ | .22^**^ | .08 | .05 | .22^**^ | 1 | .09 | -.14^+^ | -.17^*^ | -.32^***^ | -.13 |
| 7. Someone to count | -.09 | -.03 | .05 | -.12 | -.03 | -.06 | 1 | .06 | .001 | -.17^+^ | .17^*^ |
| 8. Unexpected loss | -.08 | -.12 | -.03 | .03 | -.07 | -.15^+^ | .10 | 1 | .30^***^ | .18^*^ | .14 |
| 9. Time to overcome loss | .05 | -.03 | -.04 | -.07 | -.24^**^ | -.18^*^ | -.05 | .21^**^ | 1 | .43^***^ | .26^**^ |
| 10. Depressive symptoms | -.03 | -.13 | -.21^*^ | .27^**^ | -.25^**^ | -.21^*^ | -.08 | .29^***^ | .27^**^ | 1 | .11 |
| 11. Professional help seeking | .04 | -.27^**^ | -.05 | -.12 | -.09 | -.13 | .22^**^ | .27^**^ | .29^***^ | .23^**^ | 1 |
| *Notes*. Gender: 1 = female, 2 = male. Education: 1 = compulsory school, 4 = university. Income adequacy: 1 = not enough money, 3 = more than enough money. Re-partnered: 1 = yes, 0 = no. Unexpected loss: 1 = yes, 0 = no. Time to overcome loss: 1 = less than one year, 3 = not enough time/never. Professional help seeking: 1 = yes, 0 = no. ^+^*p* < .01, **p* < .05, ***p* < .01, ****p* < .001. | | | | | | | | | | | |

Table A2. Correlations between the person and event-related characteristics, and professional help seeking for the subsample of widowed individuals (*n* = 87).

|  | 1 | 2 | 3 | 4 | 5 | 6 | 7 | 8 | 9 | 10 | 11 |
| --- | --- | --- | --- | --- | --- | --- | --- | --- | --- | --- | --- |
| 1. Age | 1 |  |  |  |  |  |  |  |  |  |  |
| 2. Gender | .24^*^ | 1 |  |  |  |  |  |  |  |  |  |
| 3. Education | -.16 | .21^+^ | 1 |  |  |  |  |  |  |  |  |
| 4. Income adequacy | -.14 | -.09^*^ | .28^**^ | 1 |  |  |  |  |  |  |  |
| 5. Time since loss | -.06 | -.05 | .04 | .06 | 1 |  |  |  |  |  |  |
| 6. Re-partnered | -.22^*^ | .32^**^ | .22^*^ | .10 | .15 | 1 |  |  |  |  |  |
| 7. Someone to count | .06 | -.14 | -.10 | .22^*^ | .03 | -.15 | 1 |  |  |  |  |
| 8. Unexpected loss | -.28^*^ | .01 | -.27^*^ | -.16 | -.09 | .06 | -.16 | 1 |  |  |  |
| 9. Time to overcome loss | -.24^*^ | -.06 | .03 | -.04 | -.17 | -.22^*^ | .06 | .16 | 1 |  |  |
| 10. Depressive symptoms | -.07 | -.09 | -.10 | .16 | -.21^+^ | -.25^*^ | -.23^+^ | .08 | .35^**^ | 1 |  |
| 11. Professional help seeking | -.20^+^ | -.19^+^ | -.05 | .09 | .14 | -.01 | -.05 | -.02 | .20^+^ | .35^**^ | 1 |
| *Notes*. Gender: 1 = female, 2 = male. Education: 1 = compulsory school, 4 = university. Income adequacy: 1 = not enough money, 3 = more than enough money. Re-partnered: 1 = yes, 0 = no. Unexpected loss: 1 = yes, 0 = no. Time to overcome loss: 1 = less than one year, 3 = not enough time/never. Professional help seeking: 1 = yes, 0 = no. ^+^*p* < .01, **p* < .05, ***p* < .01, ****p* < .001 | | | | | | | | | | | |
